# How Can the Typhoid Fever Surveillance in Africa and the Severe Typhoid Fever in Africa Programs Contribute to the Introduction of Typhoid Conjugate Vaccines?

**DOI:** 10.1093/cid/ciz629

**Published:** 2019-10-30

**Authors:** Hyon Jin Jeon, Justin Im, Andrea Haselbeck, Marianne Holm, Raphaël Rakotozandrindrainy, Abdramane Soura Bassiahi, Ursula Panzner, Ondari D Mogeni, Hye Jin Seo, Octavie Lunguya, Jan Jacobs, Iruka N Okeke, Mekonnen Terferi, Ellis Owusu-Dabo, Gordon Dougan, Megan Carey, A Duncan Steele, Jerome H Kim, John D Clemens, Jason R Andrews, Se Eun Park, Stephen Baker, Florian Marks

**Affiliations:** Department of Medicine, University of Cambridge, United Kingdom; International Vaccine Institute, Seoul, Republic of Korea; International Vaccine Institute, Seoul, Republic of Korea; International Vaccine Institute, Seoul, Republic of Korea; International Vaccine Institute, Seoul, Republic of Korea; University of Antananarivo, Madagascar; Institut Supérieur des Sciences de la Population, Université Ouaga II, Ouagadougou, Burkina Faso; International Vaccine Institute, Seoul, Republic of Korea; International Vaccine Institute, Seoul, Republic of Korea; International Vaccine Institute, Seoul, Republic of Korea; Institut National de Recherche Biomédicale, Kinshasa, Democratic Republic of Congo; Department of Clinical Sciences, Institute of Tropical Medicine, Antwerp; Department of Microbiology and Immunology, Katholieke Universiteit Leuven, Leuven, Belgium; Faculty of Pharmacy, University of Ibadan, Nigeria; Armauer Hansen Research Institute, ALERT Campus, Addis Ababa, Ethiopia; School of Public Health, Kwame Nkrumah University of Science and Technology, Kumasi, Ghana; Department of Medicine, University of Cambridge, United Kingdom; Bill & Melinda Gates Foundation, Seattle, Washington; Bill & Melinda Gates Foundation, Seattle, Washington; International Vaccine Institute, Seoul, Republic of Korea; icddr,b, Dhaka, Bangladesh; Fielding School of Public Health, University of California, Los Angeles; Korea University School of Medicine, Seoul; Stanford University, California; International Vaccine Institute, Seoul, Republic of Korea; Oxford University Clinical Research Unit, Ho Chi Minh City, Vietnam; Department of Medicine, University of Cambridge, United Kingdom; Oxford University Clinical Research Unit, Ho Chi Minh City, Vietnam; Department of Medicine, University of Cambridge, United Kingdom; International Vaccine Institute, Seoul, Republic of Korea

**Keywords:** vaccine introduction, typhoid fever, *Salmonella* Typhi, mass campaign, Ghana, Madagascar

## Abstract

**Background:**

The World Health Organization now recommends the use of typhoid conjugate vaccines (TCVs) in typhoid-endemic countries, and Gavi, the Vaccine Alliance, added TCVs into the portfolio of subsidized vaccines. Data from the Severe Typhoid Fever in Africa (SETA) program were used to contribute to TCV introduction decision-making processes, exemplified for Ghana and Madagascar.

**Methods:**

Data collected from both countries were evaluated, and barriers to and benefits of introduction scenarios are discussed. No standardized methodological framework was applied.

**Results:**

The Ghanaian healthcare system differs from its Malagasy counterpart: Ghana features a functioning insurance system, antimicrobials are available nationwide, and several sites in Ghana deploy blood culture–based typhoid diagnosis. A higher incidence of antimicrobial-resistant *Salmonella* Typhi is reported in Ghana, which has not been identified as an issue in Madagascar. The Malagasy people have a low expectation of provided healthcare and experience frequent unavailability of medicines, resulting in limited healthcare-seeking behavior and extended consequences of untreated disease.

**Conclusions:**

For Ghana, high typhoid fever incidence coupled with spatiotemporal heterogeneity was observed. A phased TCV introduction through an initial mass campaign in high-risk areas followed by inclusion into routine national immunizations prior to expansion to other areas of the country can be considered. For Madagascar, a national mass campaign followed by routine introduction would be the introduction scenario of choice as it would protect the population, reduce transmission, and prevent an often-deadly disease in a setting characterized by lack of access to healthcare infrastructure. New, easy-to-use diagnostic tools, potentially including environmental surveillance, should be explored and improved to facilitate identification of high-risk areas.

The World Health Organization (WHO) prequalified a typhoid conjugate vaccine (TCV) in December 2017, recommending its use in persons residing in typhoid fever–endemic areas [1]. In addition, TCVs have also been included in the portfolio of subsidized vaccines managed by Gavi, the Vaccine Alliance [2]. Presently, 37 Gavi-eligible countries are located in sub-Saharan Africa [3]. With the Gavi funding, a prequalified TCV, and a WHO recommendation [4], the door is open for countries to apply for supportive funds for TCV introduction. In their recent position paper, the WHO recommends the introduction of TCV in high-burden countries or areas with high presence of antimicrobial-resistant *Salmonella enterica* serovar Typhi [1]. Optimal introduction strategies, such as whether or not to conduct catch-up campaigns, and if so, which age groups, risk strata, or areas to target, are not clearly defined and should be informed by local country data. This structure potentially provides challenges for some countries, in particular resource-poor countries, where data on typhoid fever disease incidence are not readily available and where capacity to obtain these data is minimal.

## The TSAP and SETA Programs

Since 2010, the International Vaccine Institute has, in collaboration with a large number of partners, conducted the Typhoid Fever Surveillance in Africa Program (TSAP) [5] and is currently conducting the Severe Typhoid Fever in Africa (SETA) program (see Park et al in this supplement). In TSAP, thirteen study sites in ten sub-Saharan African countries were selected to conduct standardized surveillance for febrile diseases across sites to determine the incidence of typhoid fever. Given the absence of an easy-to-use diagnostic tool for typhoid fever diagnosis, blood culture–based assays were used for bacterial pathogen diagnosis, and healthcare utilization surveys were conducted to enable population-based incidence calculations [5]. Blood culture has a known sensitivity of approximately 40%–60% if procedures are followed robustly [6, 7]. While establishing TSAP, the consortium identified a number of crucial elements critical to better typhoid fever diagnosis. First, a well-established laboratory with adequately trained microbiologists and a solid quality management system were paramount to ensure appropriate diagnosis of bacterial pathogens. Second, a large proportion of contamination occurred during phlebotomy; in some sites the contamination rates exceeded 25%. Major training efforts and external monitoring were continuously necessary to ensure adherence to standardized protocol guidelines and standard operating procedures. Another challenge encountered was establishing an adequate “catchment area”—one large enough to increase likelihood of detecting typhoid cases around a surveillance site but small enough to ensure thorough case capture and understanding of healthcare-seeking behavior [5, 8]. TSAP has been successfully completed and derived standardized incidence data from sites across Africa [9]. Several important considerations are necessary to determine potential TCV introduction scenarios. Of note, TSAP showed a significant incidence in rural areas, which was similar to or, in some instances, higher than that of urban settlements. This observation could be explained by better water quality, hygiene, and sanitation infrastructure within city boundaries, as well as better access to healthcare. However, this observation needs to be confirmed in other areas, particularly owing to the temporal variability of typhoid fever incidence, as described in the Ghanaian and Kenyan TSAP sites [9, 10].

Following TSAP, the SETA program was instituted in six countries: Burkina Faso, Ghana, Nigeria, Democratic Republic of Congo (DRC), Madagascar, and Ethiopia (see Park in this supplement). Sites identified as having high typhoid fever incidence in TSAP (in Burkina Faso, Ghana, and Madagascar) were selected to continue surveillance, and new sites in three of sub-Saharan Africa’s most populous countries, Ethiopia, Nigeria, and the DRC, were established. SETA was primarily designed to investigate the incidence of severe typhoid fever, including the incidence of intestinal perforations, a commonly observed and often fatal complication of typhoid fever [11–14]. While several SETA sites exhibit a high incidence estimate of severe typhoid cases and perforations, they appear to be a function of delayed healthcare-seeking behavior. This is a particularly important finding when discussing the widescale TCV introduction in public health programs.

## The Ghana Scenario

Ghana, located in West Africa, ranks at position 140 in the Human Development Index, with an average life expectancy of 63 years and a gross national income (GNI) per capita of $4096 per year [15]. Approximately one-fifth (22%) of the Ghanaian population does not have access to safe water and relies on surface water; two-thirds of the population do not have access to improved sanitation/toilet facilities [16]. Surveillance for typhoid fever has been ongoing in Agogo, a village 80 km northeast of Kumasi, since 2008, initially within the Agogo Presbyterian Hospital and later extended to additional sites in Kumasi [9, 10]. Ghana has a functioning healthcare system in place, and the Ghanaian government has prioritized the expansion and strengthening of primary healthcare in the past five years [17, 18]. A national health insurance scheme exists that provides equitable access and financial coverage for basic healthcare services to Ghanaians [19]. Antimicrobials are readily available and sick people routinely visit healthcare facilities knowing that affordable treatment will be available [20]. Over a period of 8 years, typhoid surveillance has been conducted in the Asante Akim North District using standardized methods as outlined in TSAP/SETA [5] (see Park et al in this supplement). Incidence rates in the <15 year age group ranged from 120/100 000 person-years of observation (PYO) in 2007–2008 [10] to 389/100 000 PYO between 2010 and 2014 [9]. Further stratification of data revealed almost twice as high incidence in the population residing in rural areas compared with urban areas (636/100 000 PYO vs 297/100 000 PYO). These multiyear and multisite data demonstrate how typhoid incidence varies greatly in place and time in Ghana and, hence, pose a challenge for Ghanaian stakeholders to devise an effective typhoid control strategy for the country. Further, Ghana is scheduled to phase out of Gavi support by 2022, at which point the entire immunization program will be self-financed by the Ghanaian government [21]. As a consequence, the choice of strategy will rely heavily on healthcare infrastructure and use.

## The Madagascar Scenario

Madagascar is the fourth-largest island in the world and is located in the Indian Ocean approximately 400 km off the coast of Mozambique. It is one of the least developed nations in sub-Saharan Africa, and ranks at position 161 in the Human Development Index with an average life expectancy of 66.3 years and a GNI per capita of $1358 per year [22]. Practice of open defecation is routine and only half of the population has access to safe water, with stark differences between cities and rural areas [23]. Furthermore, the country struggles with a dysfunctional healthcare system [24]. The system is underfinanced and understaffed and essential medicines are not in place, thus, cumulating in a massive underuse of healthcare services in Madagascar [24–27]. Even for severe illnesses, people frequently succumb to disease at home since the expectation to receive healthcare at the often-distant healthcare facility is low, and families cannot afford hospital costs [24–27]. While local data on typhoid fever outcomes are sparse, historical data from the preantibiotic era demonstrated that approximately 15% of individuals with typhoid died in the absence of treatment [28, 29]; in areas of Madagascar where access to healthcare is limited, such outcomes may occur in the present day.

Typhoid fever surveillance has been put in place in two sites, one in the capital of Antananarivo and one in Imerintsiatosika, a rural area 40 km west of the capital [5]. In the capital, a state-run water supplier, Jirama, provides water at a variety of secondary outlets; hence, the majority of the population has access to microbiologically safe water. In Imerintsiatosika, however, several houses share water sources, which are often unprotected boreholes. From 2010 to 2014, the TSAP program generated incidence rates of 95/100 000 PYO and 42/100 000 PYO in Imerintsiatosika (rural) and the Isotry district in Antananarivo (urban), respectively [9], confirming a significant incidence of typhoid fever in rural areas. Notably, surveillance efforts in Madagascar have improved throughout the years, and typhoid fever remains an issue in Imerintsiatosika [25]. Nevertheless, limited data prevent appropriate assessment of typhoid fever disease incidence in other areas in Madagascar, and the Malagasy government lacks the spatially granular data to pursue a vaccination strategy targeting high-risk areas.

## Potential Vaccine Introduction Scenarios

The scenarios described in Ghana and Madagascar show that decision making for potential TCV introduction may be highly complex and multifactorial. Decision making on how best to target areas and populations for TCV introduction is largely left with the country, and no guidelines have been developed, to date, to help countries to navigate these questions. Indeed, there is no consensus as to what data are appropriate to confirm the presence and incidence of typhoid fever except blood culture–based surveillance. This is further exacerbated by the fact that there is currently only one WHO-prequalified TCV on the market, which could potentially result in a temporary supply shortfall and a need to prioritize vaccine disbursement.

We note at least two major challenges pertinent to TCV deployment, which could be addressed at the international level: diagnostic testing and regional/national introduction.

### Diagnostic Testing

During TSAP/SETA, blood culture–based diagnosis was established in all sites. This was a Herculean task, as quality typhoid diagnosis did not solely depend on the presence of a well-equipped microbiological laboratory, but also on the availability of skilled phlebotomists and technicians, contextual factors such as steady availability of materials and electricity, and other extraneous conditions relating to obtaining the sample from the patient, often in impoverished settings and with comorbidities. In some sites, the contamination rate was >10% (Mogeni et al in this supplement), resulting in a loss of data. For decision making on TCV introduction, it is probably not essential to capture every case, but obtaining valid and representative data through blood culture to confirm the presence of typhoid fever is paramount for in-country decision making and for supranational bodies to support any introduction decision. Establishing systematic, national blood culture–based surveillance is not likely possible in the near future for many low- and middle-income countries, and this has important consequences for estimating burden across varying geographies and population structures within each of Africa’s many countries.

Other diagnostic tests, such as the Widal test or rapid diagnostic tests, lack adequate sensitivity and more critically, specificity, and are therefore insufficient for assessing the actual incidence of typhoid fever. A recent publication from Ajibola et al summarizes existing diagnostic tests [30]; at this time, blood culture remains the best available tool for diagnosis and surveillance. While there might also be a significant typhoid fever incidence in African settings beyond large settlements [31], we need to consider alternative methodologies to determine the presence of *Salmonella* Typhi in the community. Two more scalable options might be using environmental surveillance to detect the presence of *S.* Typhi in sewage, implying community-level transmission, or serological surveys to determine age-related exposure to typhoid fever. However, such methods are, to date, not readily available and will need to be further developed, optimized, and validated. Without the ability to assess the population in remote settings, we will not be successful in conducting subregional or subnational campaigns directed to areas of high typhoid transmission and disease.

### Regional or National Introduction

Data from Ghana and Madagascar, as well as from the other TSAP/SETA sites, have identified that typhoid fever is highly focal and can vary in intensity, temporality, and between adjacent settings. This highlights the need for countries to examine what local data are available, assess regional data from countries with similar characteristics and identified risk factors, and utilize novel technologies and modeling to help direct optimal TCV deployment where possible. Current global recommendations are to consider the inclusion of a single TCV dose at 9 months of age with measles-rubella vaccine, and furthermore, where endemic disease transmission is high, to consider a “catch-up” campaign in all children aged ≤15 years. All costs associated with these campaigns would be supported by Gavi. In settings with high circulation of antimicrobial-resistant (AMR) *Salmonella* Typhi [32], large-scale typhoid vaccination would, beyond preventing typhoid fever in the target population, contribute substantially to reducing the spread of AMR *Salmonella* Typhi [33, 34].

For a country like Ghana, data are available from multiple sites across the country and patients having easy access to antimicrobials, an initial mass campaign introduction of TCV in high-risk areas of the country first could be considered by the National Immunization Technical Advisory Group, before expanding it to other areas in the country that have no data available. Routine introduction should follow immediately in areas for new birth cohorts after the mass campaigns. However, given the spatiotemporal heterogeneity of typhoid fever incidence, an area-targeted approach may not have any advantage over a stepwise countrywide introduction.

For countries exhibiting a similar scenario as the Republic of Madagascar, where incidence data are sparse and the healthcare infrastructure is weak, a one time national immunization campaign targeting all children <15 years of age followed by routine immunizations for new birth cohorts might be the most powerful public health approach to protect the poorest who would otherwise be at increased risk of acquiring and suffering of the consequences of the infection, including dying at home if the typhoid fever illness progresses without medical intervention. Given that Madagascar is an island with limited in- and outmigration, this could also result in the potential elimination of typhoid fever over time, a proof of concept that could be expanded to other geographically isolated areas in the future. Although circulating AMR *Salmonella* Typhi strains are not a major issue yet [9, 32], this could help to prevent the introduction and circulation of such strains into the population.

## CONCLUSIONS

The TSAP/SETA programs, and other currently ongoing typhoid fever surveillance programs in Asia, have shown that typhoid fever disease incidence is characterized by major spatiotemporal variation [9, 35]. Assessing the epidemiology of typhoid fever in endemic countries poses an important challenge to national decision makers. Blood culture–based surveillance is not easily implemented in remote settings, as was highlighted in the TSAP and SETA programs. Many countries still use the Widal test, which has poor sensitivity and specificity [7, 30], and inappropriate use reduces the validity of results even further. This has had the unfortunate consequence that governments do not believe that they have reliable data and are not moved to act.

We have now entered a new era in which TCVs are ready for deployment with Gavi support. Large, ongoing vaccination trials are in place for the assessment of indirect (herd) protection and long-term effectiveness that will determine optimal coverage rates and long-term immunogenicity and effectiveness of TCVs [36, 37]. While newly developed assays, such as the typhoid and paratyphoid test (TPTest) developed at icddr,b in Dhaka, Bangladesh [7, 38], the polymerase chain reaction–based enrichment assay developed by the Fondation Mérieux in France (unpublished), and other novel tests under development are not yet ready for introduction into the broader clinical setting, blood culture–based diagnostics remain the mainstay in typhoid diagnosis. Highly sensitive, specific, and easy-to-use high-throughput diagnostic tools coupled with environmental surveillance confirming the presence of *Salmonella* Typhi are not yet available, and it will take time until those are ready for use. In the meantime, laboratories in endemic countries that can conduct blood culture–based diagnostics should be supported and receive training to determine disease incidence and develop country-specific interventions, consequently protecting the populations in endemic countries against this deadly disease.

Data originating from blood culture–based surveillance confirming presence of *Salmonella* Typhi should, when available, be used to support decision making on TCV deployment in endemic countries and Gavi applications for TCV introduction support. Those could be combined with modeled data based on risk factors associated with typhoid fever and incidence data from neighboring countries. A larger-scale TCV introduction, consisting of a mass campaign followed by routine use, should be considered at this point given the difficulties of identifying high-risk areas with sufficient resolution and evidence. This strategy has also proven to be most cost-effective [39]. This will not only reduce the spread of *Salmonella* Typhi AMR strains, prevent the introduction of AMR strains into new areas, and keep antibiotics effective for longer periods of time, but will also protect the population from typhoid fever, particularly those with limited access to appropriate healthcare.

## Notes

***Financial support*.** This research was funded by the Bill & Melinda Gates Foundation [OPP1127988]. The International Vaccine Institute acknowledges its donors including the Republic of Korea and the Swedish International Development Cooperation Agency. This publication was made possible through a grant from the Bill & Melinda Gates Foundation [OPP1201031].

***Supplement sponsorship*.** This article was published as part of the supplement “Severe Typhoid Fever in Africa (SETA) Program” sponsored by the International Vaccine Institute.

***Potential conflicts of interest*.** All authors report no potential conflicts of interest. All authors have submitted the ICMJE Form for Disclosure of Potential Conflicts of Interest. Conflicts that the editors consider relevant to the content of the manuscript have been disclosed.

## References

[1] World Health Organization. Typhoid vaccines: WHO position paper—March 2018. Wkly Epidemiol Rec 2018; 93:153–72.

[2] Gavi, the Vaccine Alliance. Millions of children set to be protected against typhoid fever. 2017. Available at: https://www.gavi.org/library/news/press-releases/2017/millions-of-children-set-to-be-protected-against-typhoid-fever/. Accessed 31 July 2019.

[3] Gavi, the Vaccine Alliance. Countries approved for support. 2018. Available at: https://www.gavi.org/results/countries-approved-for-support/. Accessed 16 June 2019.

[4] World Health Organization. Typhoid vaccine prequalified. 2018. Available at: https://www.who.int/medicines/news/2017/WHOprequalifies-breakthrough-typhoid-vaccine/en/. Accessed 16 June 2019.

[5] von Kalckreuth V, Konings F, Aaby P, et al. The typhoid fever surveillance in Africa Program (TSAP): clinical, diagnostic, and epidemiological methodologies. Clin Infect Dis 2016; 62(Suppl 1):S9–S16. 26933028 10.1093/cid/civ693 PMC4772831

[6] Andrews JR, Ryan ET. Diagnostics for invasive *Salmonella* infections: current challenges and future directions. Vaccine 2015; 33(Suppl 3):C8–15. 25937611 10.1016/j.vaccine.2015.02.030 PMC4469564

[7] Islam K, Sayeed MA, Hossen E, et al. Comparison of the performance of the TPTest, Tubex, Typhidot and Widal immunodiagnostic assays and blood cultures in detecting patients with typhoid fever in Bangladesh, including using a Bayesian latent class modeling approach. PLoS Negl Trop Dis 2016; 10:e0004558. 27058877 10.1371/journal.pntd.0004558 PMC4825986

[8] Pak GD, Haselbeck AH, Seo HW, et al. The HPAfrica protocol: assessment of health behaviour and population-based socioeconomic, hygiene behavioural factors—a standardised repeated cross-sectional study in multiple cohorts in sub-Saharan Africa. BMJ Open 2018; 8:e021438. 10.1136/bmjopen-2017-021438 PMC6303690 30573477

[9] Marks F, von Kalckreuth V, Aaby P, et al. Incidence of invasive *Salmonella* disease in sub-Saharan Africa: a multicentre population-based surveillance study. Lancet Glob Health 2017; 5:e310–23. 28193398 10.1016/S2214-109X(17)30022-0 PMC5316558

[10] Marks F, Adu-Sarkodie Y, Hünger F, et al. Typhoid fever among children, Ghana. Emerg Infect Dis 2010; 16:1796–7. 21029549 10.3201/eid1611.100388 PMC3294512

[11] Eustache JM, Kreis DJ Jr. Typhoid perforation of the intestine. Arch Surg 1983; 118:1269–71. 6639337 10.1001/archsurg.1983.01390110027007

[12] van Basten JP, Stockenbrügger R. Typhoid perforation. A review of the literature since 1960. Trop Geogr Med 1994; 46:336–9. 7892698

[13] Parry CM, Hien TT, Dougan G, White NJ, Farrar JJ. Typhoid fever. N Engl J Med 2002; 347:1770–82. 12456854 10.1056/NEJMra020201

[14] Butler T, Knight J, Nath SK, Speelman P, Roy SK, Azad MA. Typhoid fever complicated by intestinal perforation: a persisting fatal disease requiring surgical management. Rev Infect Dis 1985; 7:244–56. 3890097 10.1093/clinids/7.2.244

[15] United Nations Development Programme. Human development reports, Ghana. Available at: http://hdr.undp.org/en/countries/profiles/GHA. Accessed 16 June 2019.

[16] Water.org. Ghana’s water and sanitation crisis. Available at: https://water.org/our-impact/ghana/. Accessed 16 June 2019.

[17] Awoonor-Williams JK, Sory EK, Nyonator FK, Phillips JF, Wang C, Schmitt ML. Lessons learned from scaling up a community-based health program in the Upper East Region of northern Ghana. Glob Health Sci Pract 2013; 1:117–33. 25276522 10.9745/GHSP-D-12-00012 PMC4168550

[18] Dalaba MA, Welaga P, Matsubara C. Cost of delivering health care services at primary health facilities in Ghana. BMC Health Serv Res 2017; 17:742. 29149853 10.1186/s12913-017-2676-3 PMC5693519

[19] Government of Ghana. National health insurance scheme. 2018. Available at: http://www.nhis.gov.gh. Accessed 31 December 2018.

[20] Krumkamp R, Sarpong N, Kreuels B, et al. Health care utilization and symptom severity in Ghanaian children—a cross-sectional study. PLoS One 2013; 8:e80598. 24244698 10.1371/journal.pone.0080598 PMC3828249

[21] Gavi, the Vaccine Alliance. Gavi, program bulletin October 2015. Available at: https://www.gavi.org/library/publications/gavi-bulletin/programme-bulletin- october-2015. Accessed 31 July 2019.

[22] United Nations Development Programme. Human development reports. Ghana. Available at: http://hdr.undp.org/en/countries/profiles/MDG. Accessed 16 June 2019.

[23] Water Aid Madagascar. Country programme strategy 2016–2021. Available at: https://washmatters.wateraid.org/sites/g/files/jkxoof256/files/WaterAid_Madagascar_Country_Programme_Strategy_2016_2021.pdf. Accessed 16 June 2019.

[24] Marks F, Rabehanta N, Baker S, et al. A way forward for healthcare in Madagascar? Clin Infect Dis 2016; 62(Suppl 1):S76–9. 26933025 10.1093/cid/civ758

[25] Marks F, Kim JH, Rakotozandrindrainy R. Madagascar should introduce typhoid conjugate vaccines now. Lancet 2018; 392:1309–10. 10.1016/S0140-6736(18)31882-8 30322578

[26] Makoni M . Madagascar’s battle for health. Lancet 2019; 393:1189–90. 30910292 10.1016/S0140-6736(19)30682-8

[27] Pach A, Warren M, Chang I, et al. A qualitative study investigating experiences, perceptions, and healthcare system performance in relation to the surveillance of typhoid fever in Madagascar. Clin Infect Dis 2016; 62(Suppl 1):S69–75. 26933024 10.1093/cid/civ892

[28] Typhoid in the largest cities of the United States 1913. JAMA 1914; 62:1473–5.

[29] Levine MM, Simon R. The gathering storm: is untreatable typhoid fever on the way? MBio 2018; 9. pii:e00482-18. 29559573 10.1128/mBio.00482-18 PMC5874914

[30] Ajibola O, Mshelia MB, Gulumbe BH, Eze AA, et al Typhoid fever diagnosis in endemic countries: a clog in the wheel of progress? Medicina (Kaunas) 2018; 54. doi:10.3390/medicina54020023. PMC6037256 30344254

[31] Saha S, Tanmoy AM, Andrews JR, et al. Evaluating PCR-based detection of *Salmonella* Typhi and Paratyphi A in the environment as an enteric fever surveillance tool. Am J Trop Med Hyg 2019; 100:43–6. 30426919 10.4269/ajtmh.18-0428 PMC6335896

[32] Park SE, Pham DT, Boinett C, et al. The phylogeography and incidence of multi-drug resistant typhoid fever in sub-Saharan Africa. Nat Commun 2018; 9:5094. 30504848 10.1038/s41467-018-07370-z PMC6269545

[33] Andrews JR, Qamar FN, Charles RC, Ryan ET. Extensively drug-resistant typhoid—are conjugate vaccines arriving just in time? N Engl J Med 2018; 379:1493–5. 30332569 10.1056/NEJMp1803926

[34] Andrews JR, Baker S, Marks F, et al. Typhoid conjugate vaccines: a new tool in the fight against antimicrobial resistance. Lancet Infect Dis 2019; 19:e26–30. 30170987 10.1016/S1473-3099(18)30350-5

[35] Cruz Espinoza LM, Nichols C, Adu-Sarkodie Y, et al. Variations of invasive *Salmonella* infections by population size in Asante Akim North Municipal, Ghana. Clin Infect Dis 2016; 62(Suppl 1):S17–22. 26933015 10.1093/cid/civ787

[36] Meiring JE, Gibani M; TyVAC Consortium Meeting Group. The Typhoid Vaccine Acceleration Consortium (TyVAC): vaccine effectiveness study designs: accelerating the introduction of typhoid conjugate vaccines and reducing the global burden of enteric fever. Report from a meeting held on 26-27 October 2016, Oxford, UK. Vaccine 2017; 35:5081–8. 28802757 10.1016/j.vaccine.2017.08.001

[37] European Developing Countries Clinical Trial Partnership. Effect of a novel typhoid conjugate vaccine in Africa. A multicentre study in Ghana and in the Democratic Republic of the Congo. Available at: http://www.edctp.org/projects-2/edctp2-projects/strategic-actions-supporting-large-scale-clinical-trials-2017/. Accessed 16 June 2019.

[38] Andrews JR, Khanam F, Rahman N, et al. Plasma immunoglobulin A responses against 2 *Salmonella* Typhi antigens identify patients with typhoid fever. Clin Infect Dis 2019; 68:949–55. 30020426 10.1093/cid/ciy578 PMC6399438

[39] Antillón M, Bilcke J, Paltiel AD, Pitzer VE. Cost-effectiveness analysis of typhoid conjugate vaccines in five endemic low- and middle-income settings. Vaccine 2017; 35:3506–14. 28527687 10.1016/j.vaccine.2017.05.001 PMC5462484

